# The Subgingival Microbiome of Periodontal Pockets With Different Probing Depths in Chronic and Aggressive Periodontitis: A Pilot Study

**DOI:** 10.3389/fcimb.2018.00124

**Published:** 2018-05-01

**Authors:** Meng Shi, Yiping Wei, Wenjie Hu, Yong Nie, Xiaolei Wu, Ruifang Lu

**Affiliations:** ^1^Department of Periodontology, Peking University School and Hospital of Stomatology, National Clinical Research Center for Oral Diseases, National Engineering Laboratory for Digital and Material Technology of Stomatology, Beijing Key Laboratory of Digital Stomatology, Beijing, China; ^2^Laboratory of Environmental Microbiology, Department of Energy and Resources Engineering, College of Engineering, Peking University, Beijing, China

**Keywords:** 16S rDNA, subgingival microbiome, chronic periodontitis, aggressive periodontitis, high-throughput nucleotide sequencing

## Abstract

Periodontitis is a kind of infectious disease initiated by colonization of subgingival periodontal pathogens, which cause destruction of tooth-supporting tissues, and is a predominant threat to oral health as the most common cause of loss of teeth. The aim of this pilot study was to characterize the subgingival bacterial biodiversity of periodontal pockets with different probing depths in patients with different forms of periodontitis. Twenty-one subgingival plaque samples were collected from three patients with chronic periodontitis (ChP), three patients with aggressive periodontitis (AgP) and three periodontally healthy subjects (PH). Each patient with periodontitis was sampled at three sites, at different probing depths (PDs, one each at 4 mm, 5–6 mm, and ≥ 7 mm). Using 16S rRNA gene high-throughput sequencing and bioinformatic analysis, we found that subgingival communities in health and periodontitis samples largely differed. Meanwhile, *Acholeplasma, Fretibacterium, Porphyromonas, Peptococcus, Treponema_2, Defluviitaleaceae_UCG_011, Filifactor*, and *Mycoplasma* increased with the deepening of the pockets in ChP, whilst only *Corynebacterium* was negatively associated with PD. In AgP, *Corynebacterium* and *Klebsiella* were positively associated with PD, while *Serratia, Pseudoramibacter, Defluviitaleaceae_UCG_011*, and *Desulfobulbus* were negatively associated with PD. And among these two groups, *Corynebacterium* shifted differently. Moreover, in subgingival plaque, the unweighted UniFrac distances between samples from pockets with different PD in the same patients were significantly lower than those from pockets within the same PD category from different patients. This study demonstrated the shift of the subgingival microbiome in individual teeth sites during disease development. Within the limitation of the relative small sample size, this pilot study shed new light on the dynamic relationship between the extent of periodontal destruction and the subgingival microbiome.

## Introduction

Periodontitis is a kind of infectious disease initiated by the colonization of subgingival periodontal pathogens, which cause destruction of the ligament and alveolar bone supporting the teeth and, ultimately, results in the loss of the affected teeth and with the resultant loss of quality of life (Newman et al., 2011; Al-Harthi et al., 2013). According to the latest official classification system for periodontal diseases from the American Academy of Periodontology, periodontitis can be classified into two main types: chronic and aggressive. Chronic periodontitis (ChP) is a slowly progressive disease, most prevalent in adults and usually associated with marked accumulation of biofilm and calculus. Conversely, aggressive periodontitis (AgP) belongs to a group of rare periodontal diseases initiated at a young age with rapid attachment loss, which is not necessarily correlated with high levels of biofilm and calculus (Armitage, 1999).

The microorganisms in a dental biofilm are believed to be involved in the pathogenesis of periodontitis; in particular, subgingival bacteria plays an important role in its initiation and progression. Decades of investigations have tried to identify a microbiological element of AgP to help in the differential diagnosis from ChP, however, the notion that AgP has a distinct microbiological pathogenesis from ChP has still not been confirmed (Armitage and Cullinan, 2010; Heller et al., 2012).

The progress of understanding oral microbiology is dependent on the development of microbial research techniques. The emergence of the next-generation sequencing (NGS) of bacterial 16S ribosomal RNA (rRNA) gene makes it possible to show a nearly unbiased view of the bacterial composition, which has the advantage of detecting non-culturable bacteria, fastidious bacteria, even novel microbes. In recent years, NGS has been widely used to analyze subgingival bacterial composition and to characterize compositional shifts between periodontal health and disease (Liu et al., 2012; Abusleme et al., 2013; Li et al., 2015; Park et al., 2015; Han et al., 2017). Some studies observed the striking differences between the microbiota of healthy sites (probing depth ≤ 3 mm) and diseased sites in ChP subjects (Ge et al., 2013; Pérez-Chaparro et al., 2018), as it is known that the composition of the subgingival microbiome varies according to probing depths possibly because of dissimilar ecological parameters such as oxygen tension (Loesche et al., 1983; Abusleme et al., 2013). However, there is scarce evidence on the shift of subgingival microbiome in individual tooth sites of both types of periodontitis during disease development.

Therefore, it is indispensable to study the microbial changes in the periodontal pockets more profoundly at the beginning and progression of different forms of periodontitis in order to provide patients with efficient preventative and treatment protocols. Thus, the main purpose of this study was using NGS of the16S RNA gene to characterize the subgingival bacterial biodiversity of pockets with different probing depths in patients with ChP or AgP, and to compare them with samples of subjects with periodontal health (PH).

## Materials and methods

### Study population and clinical examination

From January 2017 to July 2017, three patients with ChP and three patients with AgP were recruited from the Department of Periodontology, Peking University School and Hospital of Stomatology, and three PH subjects were selected as controls. The clinical inclusion criteria for all participants were that: (1) they agreed to join the research and signed an informed consent, (2) they were free of systemic disease and not pregnant or lactating, (3) they had not received any kind of periodontal treatment in the past half year, nor had taken antibiotics within the past 3 months, (4) they were non-smoker. The clinical inclusion criteria for periodontally healthy volunteers were: (1) aged from 20 to 60 years old, (2) no teeth with probing depth (PD) > 3 mm, no sties with attachment loss (AL), (3) the percentage of sites with bleeding on probing (BOP) was no more than 20%. The diagnostic criteria for AgP and ChP were based on the 1999 International Classification of Periodontal Diseases (Armitage, 1999). The clinical inclusion criteria for ChP: (1) aged from 25 to 60 years old, (2) when dividing all teeth into four quadrants (upper right, upper left, lower right, lower left), at least 2 teeth *PD* ≥ 5 mm and *AL* ≥ 3 mm in each quadrant, (3) no <20 teeth, (4) the clinical diagnosis was confirmed by full-mouth periapical radiographs. The clinical inclusion criteria for AgP were as follows: (1) aged no more than 35 years old, (2) at least 8 teeth, 3 of them not being first molars or incisors, had *PD* ≥ 5 mm and *AL* ≥ 3 mm, (3) the clinical diagnosis was confirmed by evidence of inter-proximal bone loss on full-month periapical radiographs, (4) at least 20 teeth left in mouth (Li et al., 2015).

Full-mouth clinical examinations were carried out by one practitioner. Calibration was performed on 10 patients who were not been recruited to the study, the consistency of the replicated measurements of PD and AL was recorded and calculated at the site level (Feng et al., 2015). Intraclass correlation coefficients for PD were 96% within 1 mm and AL were 90% within 1 mm. Full-mouth periodontal chartings (including PD, AL, and BOP at six sites per tooth) were been recorded and full-mouth periapical radiographs were taken as diagnostic basis.

The study protocol was reviewed and approved by the Ethics Committee of the Peking University Health Science Center (approval number: PKUSSIRB-201631135). Written informed consent was obtained from all enrolled individuals in accordance with the Declaration of Helsinki.

### Sample collection and processing

Subgingival plaque samples were collected 1 week after the full-mouth periodontal examination (Feng et al., 2015). All participants were requested to refrain from food for 8 h and oral hygiene (brushing or flossing the teeth) for 12 h before sampling. Samples were obtained in the morning (around 8 a.m. to 9 a.m.) and put on ice immediately after sampling, transported to the laboratory within 2 h and stored at −80°C before further processing (Xu et al., 2015).

The sampling method was based on Feng et al.'s research, with a little modification (Feng et al., 2015). Three subgingival plaque samples were obtained from each patient with periodontitis from mesiobuccal sites of molars, including one each with *PD* = 4 mm, 5–6 mm, and ≥ 7 mm. One subgingival plaque sample was obtained from each PH subject from the mesiobuccal site of molars with *PD* ≤ 3 mm without AL and BOP. Samples were collected individually and placed in a separate sterile 1.5 ml Eppendorf microcentrifuge tube containing 500 μl TE buffer (50 mM Tris-HCl, 1 mM EDTA; pH8.0). After isolating the selected sampling area with cotton rolls and air drying gently, supragingival plaque was removed carefully with curettes, and subgingival samples were obtained by placing a sterile Gracey curette from the apical extent of the periodontal pocket or gingival crevice and drawing it coronally with slight pressure. Clinical parameters were examined again at the sampled sites immediately after microbial sampling. The samples were centrifuged at 10,000 ×g for 10 min, and then the pellet was washed five times with 500 μl TE buffer. The samples were stored at −80°C for DNA extraction.

### Microbial DNA extraction

Genomic DNA was extracted from the subgingival plaque using a QIAamp DNA Mini Kit (QIAGEN Sciences, USA) following the manufacturer's instructions, with an extra lysozyme treatment (3 mg/ml, 1.5 h) for bacterial cell lysis. DNA concentration and purity were measured with a NanoDrop ND-1000 spectrophotometer (Thermo Fisher Scientific, USA) and monitored on 1% agarose gels. The results showed that the A260:A280 ratios were 1.8–2.1 and the DNA concentrations were all more than 20 ng/μl. According to the concentration, DNA was diluted to 1 ng/μl using sterile water. The extracts were stored at −80°C until use.

### 16S rRNA gene sequencing

The 16S rRNA V4 gene was analyzed to evaluate the bacterial composition and diversity using Illumina Hiseq (Novogene Bioinformatics Technology Co., Ltd.). All the DNA samples satisfied the quality and quantity standards of sequencing. Polymerase chain reaction(PCR) amplification of the V4 region of the bacterial 16S rRNA gene was performed using specific primers, 515F (5′, GTGCCAGCMGCCGCGGTAA, 3′) and 806R (5′, GGACTACNNGGGTATCTAAT, 3′). PCR reactions were carried out in 30 μL reactions with 15 μL of Phusion^®^ High-Fidelity PCR Master Mix (New England Biolabs, USA), 0.2 μM of forward and reverse primers, and approximately 10 ng template DNA. The PCR conditions were as follows: initial denaturation at 98°C for 1 min, followed by 30 cycles of denaturation at 98°C for 10 s, annealing at 50°C for 30 s, and elongation at 72°C for 30 s. Finally, 72°C for 5 min. The PCR products were confirmed by 2% agarose gel electrophoresis. Samples with bright main strip between 400 and 450 bp were chosen for further experiments. The PCR products were mixed in equidensity ratios. Further, mixed PCR products were purified with GeneJET Gel Extraction Kit (Thermo Fisher Scientific, USA).

Sequencing libraries were generated using TruSeq^TM^ DNA PCR-Free Sample Preparation Kit (Illumina, USA) following manufacturer's recommendations and index codes were added. The library quality was assessed on the Qubit^®^ 2.0 Fluorometer (Thermo Fisher Scientific, USA) and Agilent Bioanalyzer 2100 system (Agilent, USA). At last, the library was sequenced on an Illumina HiSeq 2,500 and 250 bp paired-end reads were generated (Yuan et al., 2018). The raw reads were deposited into the NCBI Sequence Read Archive (SRA) database (Accession Number: SRP125475).

### Bioinformatic analysis, statistical analysis, and visualization

Paired-end reads from the original DNA fragments were merged using FLASH-a very fast and accurate analysis tool, which is designed to merge paired-end reads when there are overlaps between reads1 and reads2 (Magoč and Salzberg, 2011). Paired-end reads was assigned to each sample according to the unique barcodes. Sequences were analyzed using QIIME software package (Quantitative Insights Into Microbial Ecology), and in-house Perl scripts were used to analyze alpha- (within samples) and beta- (among samples) diversity (Caporaso et al., 2010). First, reads were filtered by QIIME quality filters. Subsequently, pick_*de_novo*_otus.py was used to pick operational taxonomic units (OTUs) by making an OTU table. Sequences with ≥ 97% similarity were assigned to the same OTUs. A representative sequence was picked for each OTU and the RDP classifier was used to annotate taxonomic information for each representative sequence (Wang et al., 2007). After rarified the OTU table, two metrics were calculated for the evaluation of alpha diversity: abundance-based-coverage (ACE) estimates the species abundance; and the diversity of the sample microbiota was estimated by the Shannon index. Normality tests for each group of data were conducted. The Student's *t*-test was used to compare significant differences of the alpha diversity indexes between the different groups (*p* < 0.05). A principal component analysis (PCA) was conducted on the OTU level to evaluate the similarity of microbial community structure among various groups. The Unweighted UniFrac distances, a phylogenetic measures of beta diversity was calculated by QIIME. Differences in the Unweighted Unifrac distances for comparisons among groups were analyzed by Student's *t*-test and visualized by constructing a scatter diagram and box plot. PCA analysis, scatter diagram and box plot and heatmap were performed using R 3.2.5. The taxonomy compositions and abundances were visualized by GraphPad PRISM® software (version 4.0). To compare the taxa in subgingival plaque between PH, AgP, and ChP, the Mann–Whitney test was used. Spearman Correlation Analysis was used to find the correlation between the relative abundance of specific microorganisms in subgingival plaque and PD. The Mann–Whitney test, Student's *t*-test and Spearman Correlation Analysis were performed using SPSS 20.0.

## Results

### Increasing diversity in subgingival bacterial communities from periodontitis patients

Nine subjects were enrolled in the study, including 21 subgingival plaque samples. The demographic and clinical characteristics of subjects are depicted in Table 1. Based on the full-mouth examination, ChP and AgP had higher BOP, PD, and AL values than PH subjects. A total of 1,608,835 V4 16S rDNA paired-end reads were generated from 21 samples. After filtering, 1,242,888 reads were left. The average length of the filtered reads was 419 bp. A total of 391 OTUs were obtained at a 97% similarity level and assigned into 17 phyla, 28 classes, 52 orders, 84 families, and 166 genera.

**Table 1:** Demographic and clinical characteristics of the study population.

	**PH1**	**PH2**	**PH3**	**ChP1**	**ChP2**	**ChP3**	**AgP1**	**AgP2**	**AgP3**
Gender	F	M	F	M	F	M	F	F	F
Age	23	21	26	33	52	28	26	32	24
Number of teeth	28	28	28	28	28	27	28	28	28
**FULL-MOUTH EXAMINATION**									
PD (mm)	2.76 ± 0.73	2.47 ± 0.59	2.43 ± 0.62	4.26 ± 1.70	4.56 ± 1.60	4.22 ± 1.48	4.55 ± 1.45	4.49 ± 1.35	3.86 ± 1.14
AL (mm)	0	0	0	3.13 ± 1.97	2.76 ± 1.97	3.04 ± 3.00	2.04 ± 1.53	3.73 ± 2.32	3.03 ± 1.55
BOP (%)	17.86	7.14	14.29	100	100	100	100	100	100

*Values are means ± standard deviations, median (Q25; Q75) and numbers of participants (percentage). M, male; F, female; PD, probing depth; AL, attachment loss; BI, bleeding index; BOP, bleeding on probing*.

**Figure 1:**
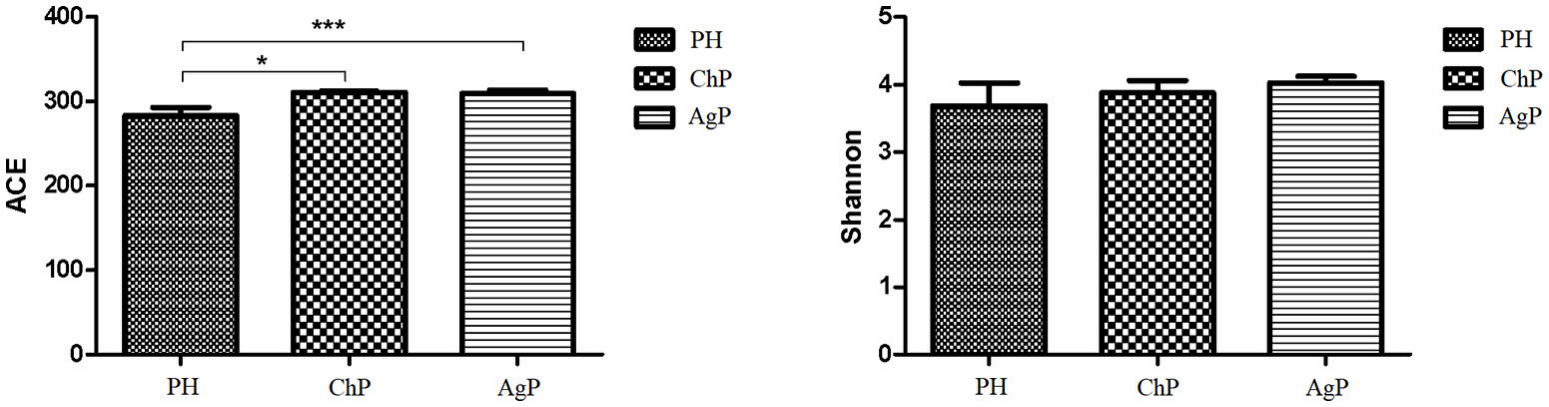
Microbiota alpha-diversity as calculated by ACE and Shannon index. The ACE index values were significantly lower in subgingival plaque samples of the health group (PH) than the chronic periodontitis group (ChP) and aggressive periodontitis group (AgP). A tendency toward an increase in the Shannon index was observed in samples of ChP/AgP compared to plaque from PH; however, the difference was not significant. ^*^p ≤ 0.05; ^***^p ≤ 0.001.

ACE showed a significant difference between ChP and PH, as well as between AgP and PH. Compared with the PH samples, the ACE index of the subgingival microbial community was significantly higher in patients with periodontitis. The Shannon index also increased in ChP and AgP, although there was no significant difference among PH, ChP, and AgP (Figure 1). The results indicated that the alpha-diversity of subgingival communities from patients with periodontitis was higher than those in healthy subjects. Neither the groups of the same kind of periodontitis with different PD, nor between the groups of different kind of periodontitis with the same PD had significant difference.

### Compositions in subgingival bacterial communities

**Figure 2:**
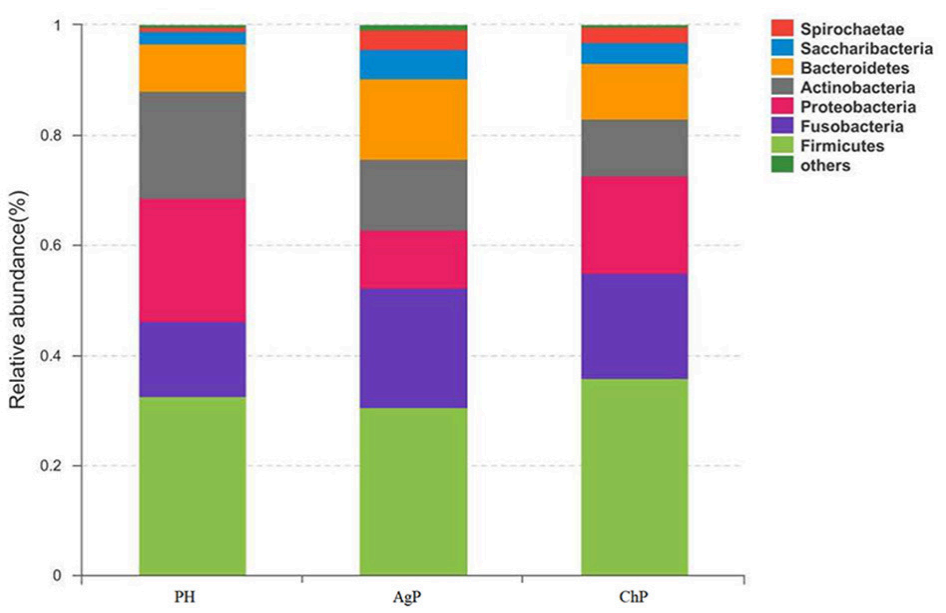
Relative abundance of bacterial composition at phylum level in health group (PH), chronic periodontitis group (ChP) and aggressive periodontitis group (AgP).

**Figure 3:**
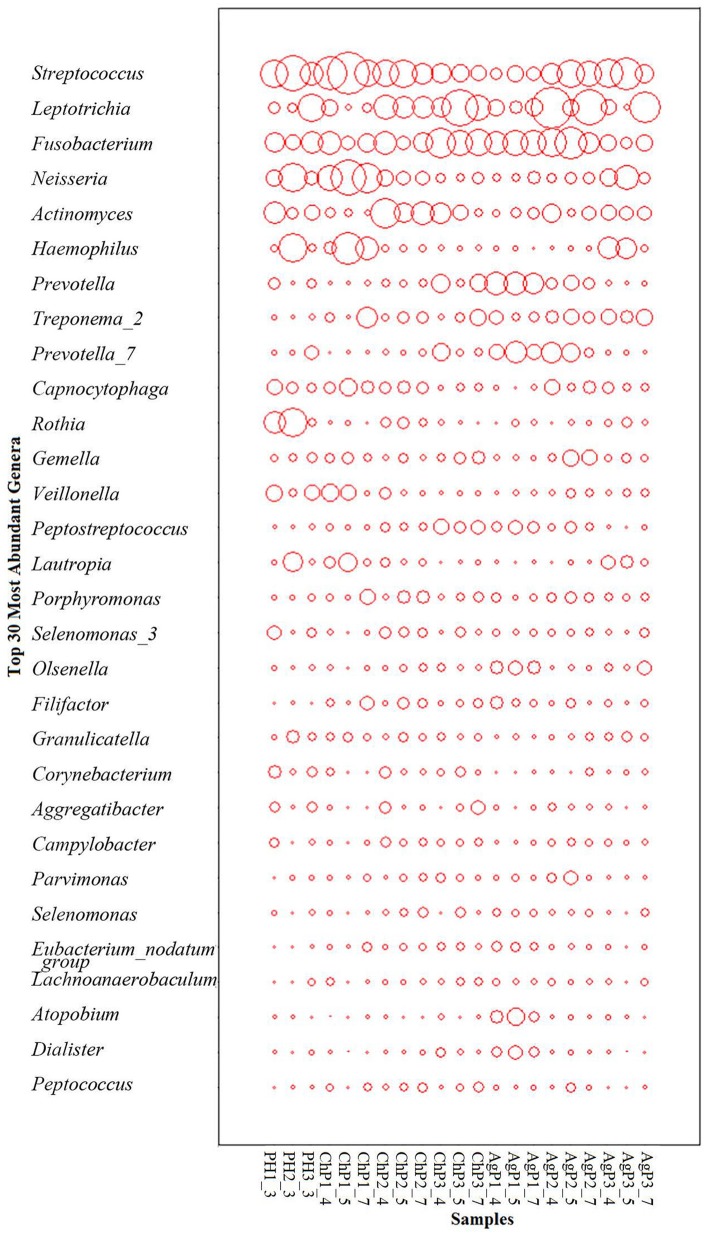
The distribution of the major genera in the microbiomes of periodontal health and disease. The y-axis shows the top 30 most abundant genera which constitute 87.06~91.63% of each bacterial microbiota. The relative abundance of each genus is indicated by the circle area.

As for taxonomic analysis, we found that, PH, ChP, and AgP were all dominated by Firmicutes (from 30.73 to 35.87%), and abundant in Proteobacteria (from 10.35 to 22.27%), Fusobacterium (from 13.69 to 21.77%), Actinobacteria (from 10.33 to 19.46%), Bacteroidetes (from 8.58 to 14.83%) at the phyla level. Compared with healthy samples, the proportion of Fusobacterium, Spirochaetae, and Saccharibacteria were increased in ChP and AgP (Figure 2). At the genus level, *Streptococcus* (10.10–16.44%)*, Leptotrichia* (6.49–11.21%)*, Fusobacterium* (7.18–10.54%)*, Neisseria* (3.84–8.43%)*, Actinomyces* (3.54–6.03%)*, Haemophilus* (2.51–6.12%)*, Prevotella* (1.83–4.71%)*, Treponema_2* (0.64–3.66%), and *Capnocytophaga* (1.85–3.15%) accounted for more than 60% of sequencing results in all subgingival samples, though there were some differences in the precise proportions between PH, ChP, and AgP (Figure 3).

### Comparison of subgingival bacterial communities between healthy and periodontitis samples

**Figure 4:**
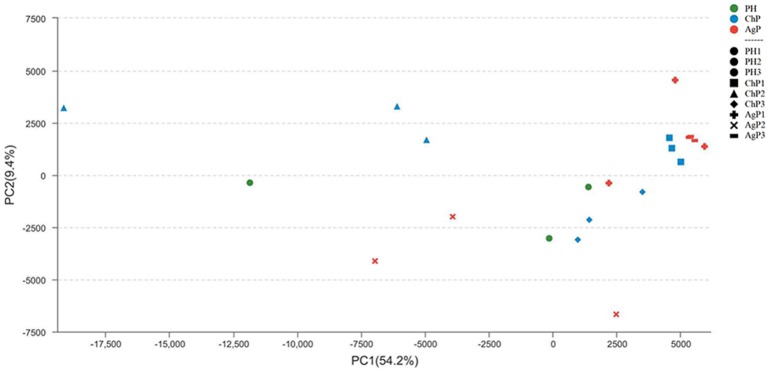
Principal component analysis at the OTU level at 97% identity. Each sample is represented by a dot. Green dots represent the samples of the health group (PH). Blue dots represent the samples of the chronic periodontitis group (ChP). Red dots represent the samples of aggressive periodontitis group (AgP). The shape of the dots represents the samples from different persons (except PH). PC1 explained 54.2% of the variation observed, and PC2 explained 9.4% of the variation. ChP and AgP clustered together more closely, while PH was more dispersive, suggesting that the bacterial structures in diseased groups were more similar.

To investigate the distribution of bacterial communities among all samples, a principal component analysis (PCA) was performed based on the OTU abundances. The ChP and AgP samples clustered together and could not be distinguished from each other, while the PH samples appeared more dispersive (Figure 4). We also noticed that among the same type of periodontitis (ChP/AgP), samples from the same patient clustered closer together than others of the same PD.

**Figure 5:**
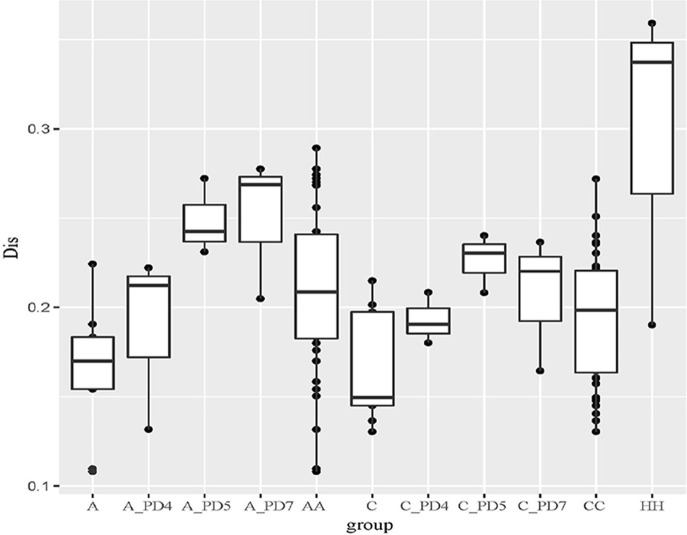
The Unweighted Unifrac distance between samples. A, distance between samples from the same AgP patients; A_PD4, distance between samples of *PD* = 4 mm sites from different AgP patients; A_PD5, distance between samples of *PD* = 5–6 mm sites from different AgP patients; A_PD7, distance between samples of *PD* ≥ 7 mm sites from different AgP patients; AA, distance between samples from different AgP patients. C, distance between samples from the same ChP patients; C_PD4, distance between samples of *PD* = 4 mm sites from different ChP patients; C_PD5, distance between samples of *PD* = 5–6 mm sites from different ChP patients; C_PD7, distance between samples of *PD* ≥ 7 mm sites from different ChP patients; CC, distance between samples from different ChP patients. HH, distance between samples from different healthy subjects.

The Unweighted UniFrac distance between periodontally healthy subjects (HH), ChP patients (CC), and AgP patients (AA) were also calculated to evaluate the relationships of all samples. As Figure 5 showed, the Unifrac distances between samples from the same individuals were significantly lower than those between samples either within the ChP or AgP groups, using Independent *t*-test, *p* = 0.046 (CC-C)/*p* = 0.008 (AA-A). It was notable that, in the subgingival plaque samples, the distances between the H subjects were higher than that between ChP patients/AgP patients, using Independent *t*-test, *p* = 0.195 (HH-CC)/*p* = 0.008 (HH-AA).

The distances between samples in the same PD group from different ChP/AgP patients were also calculated (showed in Figure 5 as C_PD4, C_PD5, C_PD7, A_PD4, A_PD5, A_PD7). Still, the distances between samples from the same ChP/AgP patients were lower than that samples in the same PD group from different ChP/AgP patients, using Independent *t*-test, *p* = 0.09 (C-C_PD4)/*p* = 0.004 (C-C_PD5)/*p* = 0.1 (C-C_PD7)/*p* = 0.374 (A- A_PD4)/*p* = 0.004 (A- A_PD5)/*p* = 0.006 (A- A_PD7). These results were consistent with the ones obtained from the comparison of healthy and periodontitis participants described above.

Although the bacterial communities were mainly constructed dependent on individuals, the abundances of specials bacteria were found significantly different between healthy and patients. 391 OTUs were measured in the PH, ChP and AgP, with 299, 303,301 361 OTUs shared by samples PH/ChP/AgP, PH/ChP, PH/AgP, ChP/AgP, respectively. The numbers of Unique OTUs in the PH, ChP, and AgP groups were 2 (belonging to *Bergeyella*), 10 (belonging to *Fastidiosipila, Vibrio, Reyranella, Rhodococcus, Propionibacterium, Treponema_2, Anaeroplasma*), and 12 (belonging to *Bacteroides, Rhodobacter, Mycoplasma, Devosia, Treponema_2, Prevotella, Bacillus*), respectively.

**Figure 6:**
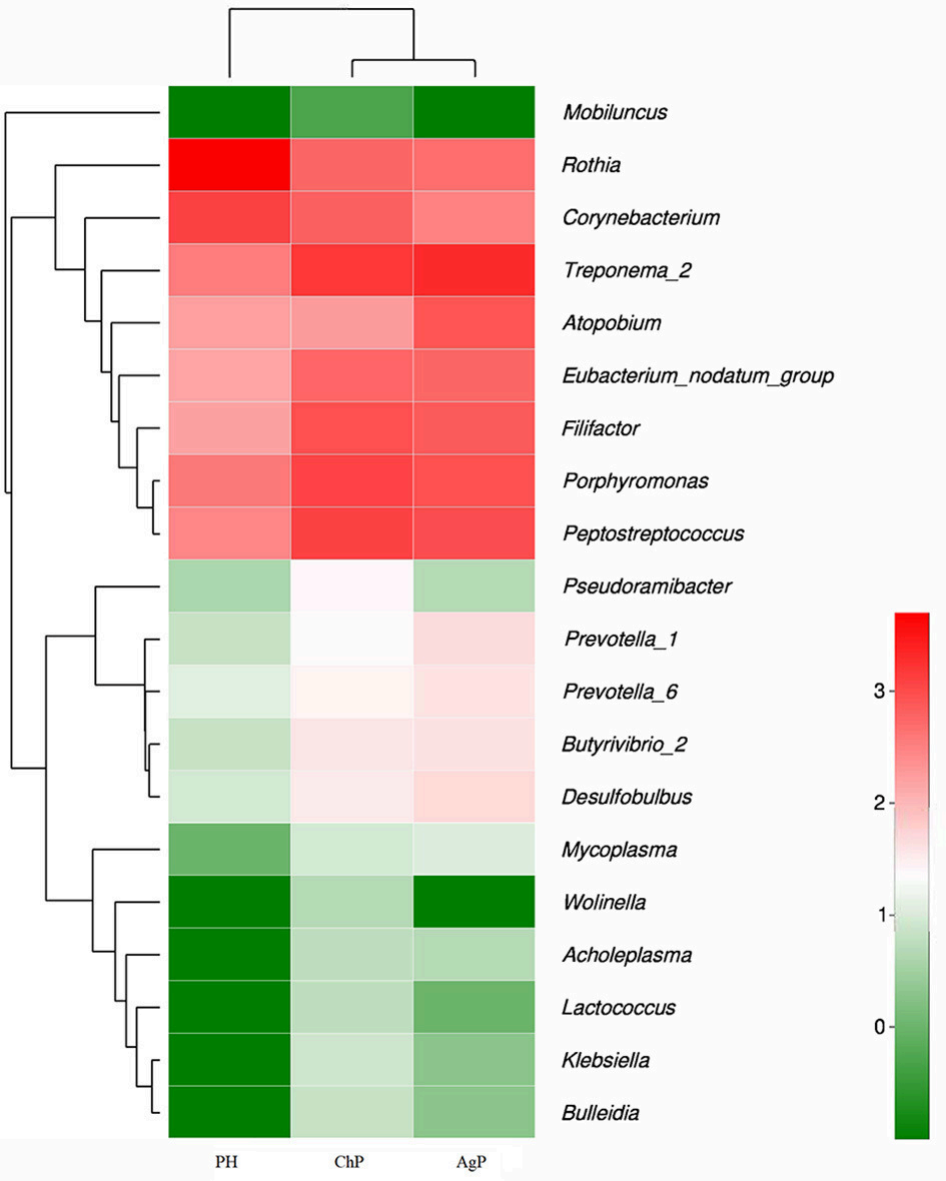
Heatmap presenting the distribution of the genera which showed significant differences in relative abundance between PH, ChP, and AgP (Log2 transformed counts). Samples groups are represented in the x-axis. Genera are listed in the right y-axis, and hierarchical clusters in the left y-axis. High genera counts are represented in red and low genera counts are represented in green.

Microbiota between groups on different levels were compared by using the Mann-Whitney *U*-test. Each of the 17 phyla, 28 classes, 52 orders, 84 families, and 166 genera were compared between PH and ChP, PH and AgP, ChP and AgP. Figure 6 presents a heatmap of the genera which had significant differences between groups.

There were significant differences between the PH and ChP at genus level: the abundance of *Rothia* was significantly higher in PH, other genera such as: *Peptostreptococcus, Filifactor, Eubacterium_nodatum_group, Butyrivibrio_2, Klebsiella, Bulleidia, Lactococcus, Wolinella*, and *Acholeplasma* increased significantly in ChP.

The differences between PH and AgP at the genus level were: the abundance of Rothia, Corynebacterium, Mobiluncus significantly increased in PH; while Treponema_2, Porphyromonas; Filifactor, Eubacterium_nodatum_group, Desulfobulbus, Prevotella_1, Prevotella_6, Butyrivibrio_2, Mycoplasma, and Lactococcus were higher in AgP.

The subgingival microbiota compositions of the ChP and AgP groups were also been compared, and differences were found in the following bacteria: except *Atopobium*, other genera such as: *Pseudoramibacter, Klebsiella, Lactococcus*, and *Wolinella* were more abundant in the ChP group.

### Influence of periodontal probing depth on subgingival bacterial communities

**Figure 7:**
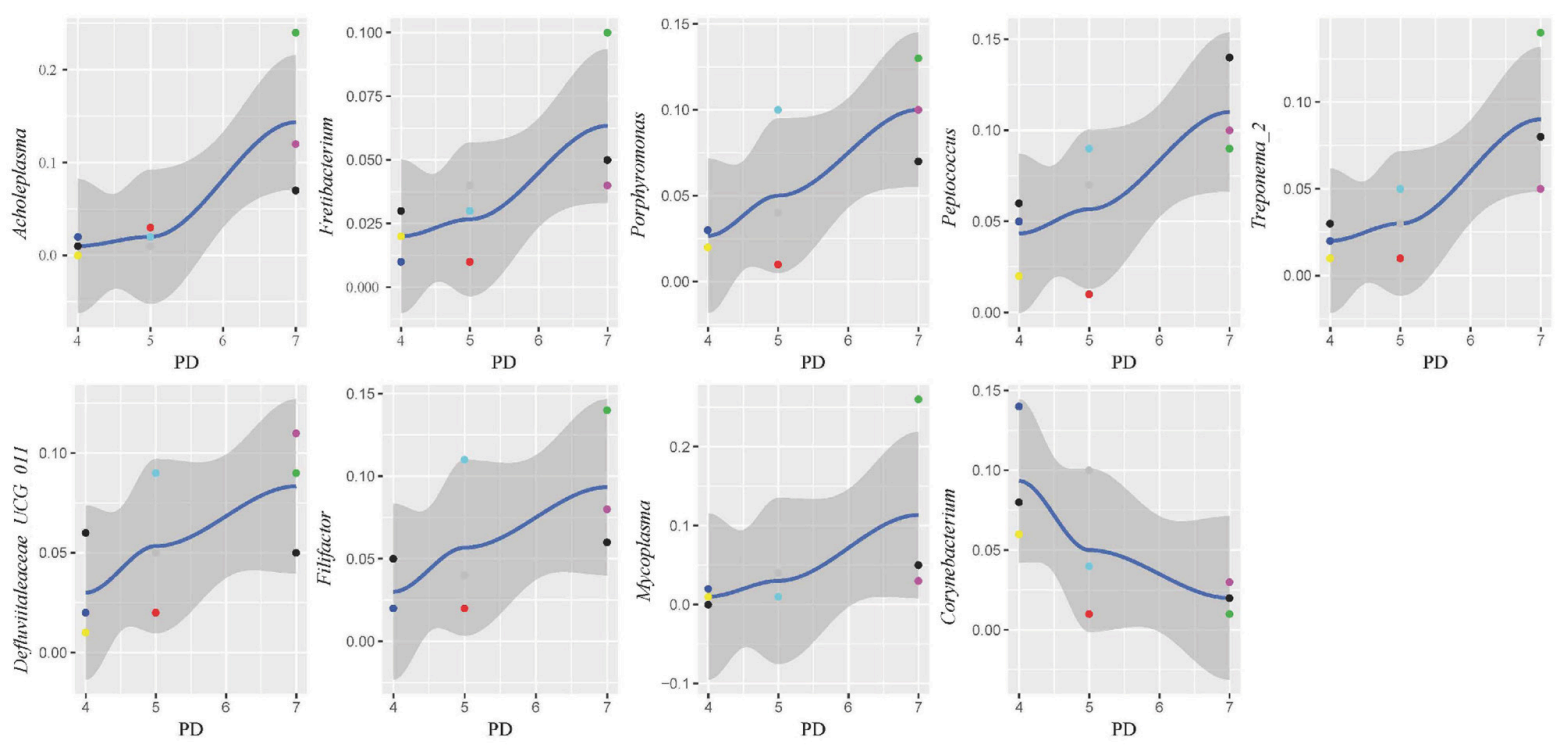
Scatter diagram of bacteria whose relative abundance were correlated significantly with the changing periodontal probing depths (PDs) in chronic periodontitis group (ChP).

**Figure 8:**
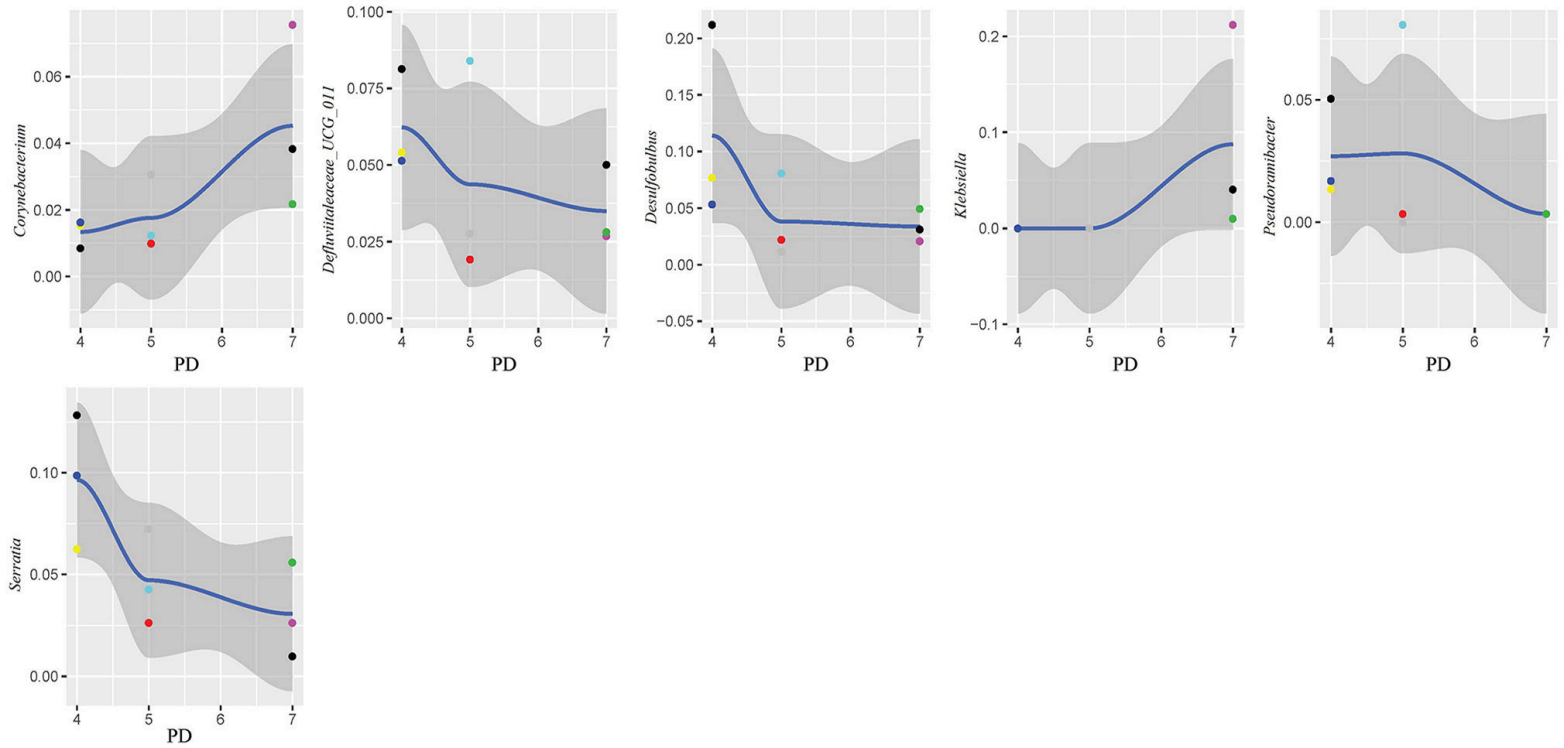
Scatter diagram of bacteria whose relative abundance were correlated significantly with the changing periodontal probing depths (PDs) in aggressive periodontitis group (AgP).

In this study, subgingival plaque samples were collected at different depth of periodontal pockets from every patient with periodontitis. Subsequently, we analyzed the Spearman correlation between the community structure of each sample at the genus level and its corresponding periodontal probing depth. The results indicated that along with the changes of PD, the relative abundance of several bacteria changed in ChP and AgP, and some among them showed significant linear correlation. In ChP, bacteria positively associated with PD were: *Acholeplasma, Fretibacterium, Porphyromonas, Peptococcus, Treponema_2*, Defluviitaleaceae*_UCG_011, Filifactor*, and *Mycoplasma*, whilst only *Corynebacterium* was negatively associated with PD. In AgP, *Corynebacterium* and *Klebsiella* were positively associated with PD, while *Serratia, Pseudoramibacter*, Defluviitaleaceae*_UCG_011*, and *Desulfobulbus* were negatively associated with PD (Figures 7, 8).

## Discussion

In our current research, we found that periodontal destruction was associated with higher alpha diversity of subgingival microbial community, combined with a shift in the composition of microbiota. The results also indicated that, along with the changes of PD of the periodontal pockets in the same periodontitis patient, the relative abundance of several bacteria also changed in both ChP and AgP, and some among the genus showed significant liner correlation. In addition, the results based on the Unweighted UniFrac distance between all the samples, showed that the subgingival bacterial communities might be mainly influenced by the whole oral environment, but not only the local diseased region.

Periodontitis is an infectious disease associated with a variety of microorganisms. For many years, the study of the etiology of bacteriology of periodontitis has continued. Comprehensive understanding of the ecology of the subgingival microbiota and the dynamic relationship between disease development and subgingival microbiome is essential for developing efficient prevention and treatment strategies for periodontitis. Using 16S rRNA gene high-throughput sequencing can give us broader insight than traditional methods, such as using PCR or real-time fluorescence quantitative PCR. Because of the advantages of high throughput, 16S rRNA gene sequencing can detect more existing microbes in test samples, providing comprehensive information on the composition and structure of the subgingival plaque microbes, as well as its changing trend with different periodontal status.

In this study, we used 16S rRNA gene sequencing to evaluate and compare the differences of the characteristics of subgingival plaque between PH, ChP, and AgP patients. The results showed that the presence of periodontitis, both ChP and AgP, were associated with higher alpha diversity of subgingival bacterial communities, which is in accordance with previous research (Griffen et al., 2012; Liu et al., 2012). This kind of increase may result from the formation and accumulation of dental plaque biofilms, which occurs at the onset of periodontitis. The increased alpha-diversity of subgingival microbiome may indicate periodontal disease status.

In the current study, because of taxonomic analysis, we found that as the health status of periodontal tissue changes, the composition of subgingival plaque community shifted, and there were also differences in bacteria associated with different types of periodontitis. Previous research showed that the subgingival microbiota of ChP patients (Griffen et al., 2012) and AgP patients (Han et al., 2017) were dominated by Firmicutes, Actinobacteria, Bacteroidetes, Fusobacteria, and Proteobacteria at the phyla level, and in addition, the proportion of Spirochaetes and Synergistetes was increased compared with healthy samples. Our study confirmed these findings. When comparing ChP and PH, *Peptostreptococcus, Eubacterium_nodatum_group, Mycoplasma*, and *Filifactor* were relatively more abundant at the genus level in ChP, which has also been reported in previous studies (Kumar et al., 2005; Li et al., 2014; Shi et al., 2015). As for the difference between AgP and PH, our result was in agreement with the study of Han et al.: *Treponema_2, Porphyromonas, Prevotella*, and *Corynebacterium* were found at increased abundance in AgP compared with PH, while *Rothia* presented at higher abundance in PH (Han et al., 2017). Among these subgingival genera, some included well-known destructive periodontal pathogens, such as *Treponema_2* (*Treponema denticola*), *Porphyromonas* (*Porphyromonas gingivalis*), *Prevotella* (*Prevotella nigrescens, Prevotella intermedia*, and *Prevotella melaninogenica*) (Socransky et al., 1998). Meanwhile, we also found that some low abundant subgingival genera played roles in the progression of periodontitis, such as *Butyrivibrio_2* and *Bulleidia*. These genera may contribute to the changes in diversity between the subgingival plaque samples of periodontally healthy subjects and periodontitis patients. In conclusion, a distinct microbiome is found in different kind of periodontitis in the present study.

The changes in the subgingival microbiome of individual tooth sites at the onset and progression of periodontitis have not been well characterized. Most of the previous studies were performed using pooled plaque samples from multiple individuals, or from multiple tooth sites of the same individuals (Wang et al., 2013; Li et al., 2014, 2015; Park et al., 2015). There has been little research sampling from different tooth sites in the same individual and then testing separately in order to explore the difference of the microbiome between tooth sites. Even in the same individual, differences of clinical conditions between sites still exist, that may lead to the structure and composition of subgingival plaque because of change in periodontal pockets. Finding the similarities and differences of the characteristics of subgingival plaque under different clinical conditions in the same individual could help us to understand the roles of microorganisms played in the pathogenesis of periodontitis. One study has compared the subgingival microbiome between shallow (periodontally healthy) and deep (periodontally diseased) pocket sites in the same ChP patients and found that there were differences in compositions (Ge et al., 2013). Meanwhile, as the periodontal pockets deepen, the physical and chemical properties of subgingival ecosystem also changed, such as pH (Forscher et al., 1954), temperature (Haffajee et al., 1992), and the oxygen tension (Mettraux et al., 1984). The pockets with same depth presented similar ecological niches. Regarding to the study of Abusleme et al., the microbiomes in the two sites with same probing depths within the same individual were more similar than those from different individuals (Abusleme et al., 2013). Based on these findings, in this study, we divided the tooth sites of periodontitis patients more finely. Each subject was sampled at three sites which differed in periodontal destruction, as evaluated by PD. The results showed that along with changing PD, the abundance of several subgingival genera changed in ChP and AgP. A small amount of genera with statistical differences, such as *Porphyromonas* and *Treponema_2*, included well-known periodontopathogens (Socransky et al., 1998), while most of the genera were nondominant which was currently been researched little. However, along with the change of PD, the trends of some subgingival genera shifts were different between ChP and AgP, and were even opposite in some cases. For instance, *Corynebacterium* had a negative correlation with PD in ChP, while the opposite occurred in AgP. *Corynebacterium* had been reported to inhabit the oral cavity and some species might be associated with the formation of dental calculi (Tsuzukibashi et al., 2014). Because of our finding, there might be different *Corynebacterium* species that play a role in ChP and AgP.

It should be noted that when analyzing data, most of the studies classified the samples from periodontal pockets which PDs were 4–6 mm as one group (Socransky et al., 1991, 1998; Haffajee and Socransky, 2001; Pérez-Chaparro et al., 2018). This kind of cluster was mainly based on the experimental results by microbial cultivation or checkerboard DNA hybridization techniques. However, some other researchers found that samples from periodontal pockets with 4 mm-depth were slightly different from those with other depth, including the counts of microbial species (Socransky et al., 1991), some specific periodontal pathogens (Eick and Pfister, 2002) and the response to periodontal treatment (Haffajee et al., 2006). Therefore, in this study, samples from periodontal pockets with 4 mm-depth were specifically classified as one group, the purpose was to analyze the comprehensive information of the composition and structure of the subgingival plaque microbes by high-throughput sequencing. Based on the limited sample size, the result showed that the structure of subgingival microbiota from periodontal pockets with 4 mm-depth were not significantly different from those with 5 and 6 mm-depth, although along with changing PD, the abundance of several subgingival genera changed.

The periodontal ecological environment is complex, and dental plaque is considered to contain high species diversity, be site-specific, and have relatively stable bacterial biofilms. The current findings suggest that more than 700 species of microorganisms have settled in the oral cavity (Aas et al., 2005). The exploration of bacterial etiology of periodontitis has undergone several stages, and generated several hypotheses/theories. In the current study, we tried to compare the differences of the subgingival microbiome between samples by using Unweighted Unifrac distance. According to the Unweighted Unifrac distance of the subgingival plaque samples from different groups, it could be concluded that the distance between samples from different PH subjects (shown as HH in Figure 5) was farthest, which meant that the structure and composition of healthy periodontal subgingival plaque was highly individual. This finding was consistent with some previous research, which indicated that the commensal or normal microbiome were diverse in different individuals, even between different tooth sites in the same individual (Shi et al., 2015; Han et al., 2017). This regularity might result from the shallow gingival sulcus, where the subgingival plaque could be easily influenced by environmental factors (for example oral hygiene habits, diets and lifestyles). Although the formation of plaque is rapid, it still takes time for a plaque biofilm to reach maturity, and because of good oral health habits, healthy individuals usually clean plaque away before maturation. Consequently, the subgingival plaque community in periodontally healthy subject could never reach a stable structure. In this case, the occurrence of periodontal pathogens would be transient and random, and have no pathogenic roles at all. This could also explain why some studies have found that periodontal pathogens are present in healthy sites of periodontal healthy individuals (Bik et al., 2010).

The distances between samples from different ChP/AgP patients in their own group were less than that with PH subjects (shown as CC, AA, and HH in Figure 5), which indicated that the composition of different ChP/AgP patients' subgingival plaque were similar, which suggested the similar succession trajectories of subgingival bacteria communities during the development of periodontitis. Although mechanical methods, for example, effective brushing, using floss, and periodontal treatment, can effectively control plaque, as the periodontitis progresses and the periodontal pocket becomes deeper, the structure of the subgingival plaque biofilm becomes more complex and difficult to control, making the course of the disease more difficult to reverse. Although ChP/AgP patients were affected by the same factors as periodontally healthy individuals, because of the formation and deepening of periodontal pockets, the influence of the oral cavity decreased gradually on subgingival plaque composition. The bacteria existing in periodontal pockets were not susceptible to saliva flushing, food friction, or oral hygiene measures (brushing teeth, gargling etc.). These conditions provide opportunities for the formation and maturation of plaque biofilms, making the bacteria related to periodontitis gradually stabilize in the periodontal pockets, playing a role in pathogenicity. These aspects are interrelated and dynamic, and have no clear cause-and-effect relationship.

In our current study, we collected 3 samples with each ChP/AgP, and compared the distances between ChP/AgP in the same extent of periodontal destruction (according to PD). The result showed that the distances between samples from pockets with different PD in the same patients were lower than those from pockets within the same PD category from different patients. This result proved that the difference of plaque structure between tooth sites from same individual was the smallest, which implied that, although different tooth sites might present varying extents and severities of periodontal destruction in the progress of periodontitis, the patient's whole periodontal microenvironment changes with similar tendencies. Griffen et al. observed that the preponderance of disease-associated organisms was found in both periodontally healthy and diseased sites of subjects with disease (Griffen et al., 2012). Both of our results suggested that the community shifts happened in all pockets, and that the structures of plaque in the same patient were similar between periodontal pockets of different depth. This indicates that any periodontal pocket might be a reservoir for periodontal pathogens, independently of its depth. This knowledge suggests that during periodontal treatment, even periodontal pocket with 4 mm need clinician to pay high attention, as the shallow pockets in patients with periodontitis may represent a transitional period in the process of the disease.

Within the limitation of the relative small sample size, the present study demonstrates the tendency of dynamic changes in the subgingival microbiome of individual tooth sites during periodontal disease development. Additional pre-clinical studies appraising virulence factors of the special genera associated with PDs and the interactions with the host and interventional studies evaluating their behavior after treatment are needed in the future study. If longitudinal sequencing data from different extents of periodontal destruction for each periodontitis subject in a larger cohort study are collected, the dynamic changes in the subgingival microbiome during disease development can be illustrated more thoroughly, which would provide a better understanding of the etiology of periodontitis.

## Conclusions

In our study, we found that subgingival communities in healthy samples and periodontitis largely differed, with higher bacterial abundance in periodontitis. Meanwhile, along with the changes of PD, the relative abundance of several bacteria changed in ChP and AgP, and some among them shifted differently between the two groups, even opposite in some cases. Moreover, in subgingival plaque, the unweighted UniFrac distances between samples from pockets with different PD in the same patients were significantly lower than those from pockets within the same PD category from different patients. Within the limitation of the relative small sample size, this pilot study shed light on the dynamic relationship between the extent of periodontal destruction and the subgingival microbiome.

## Author contributions

WH and XW conceived and designed the experiments. MS, YW, and RL performed the experiments. MS and YN analyzed the data. MS and YN contributed reagents, materials, and analysis tools. MS and YW prepared the manuscript. MS, YW, WH, XW, and RL revised the manuscript.

### Conflict of interest statement

The authors declare that the research was conducted in the absence of any commercial or financial relationships that could be construed as a potential conflict of interest.
